# Crystal structures of coordination polymers from CaI_2_ and proline

**DOI:** 10.1107/S2056989015009597

**Published:** 2015-05-23

**Authors:** Kevin Lamberts, Ulli Englert

**Affiliations:** a Institute of Inorganic Chemistry, RWTH Aachen, Landoltweg 1, 52074 Aachen, Germany

**Keywords:** crystal structure, amino acid, coordination polymer, calcium, proline

## Abstract

Completing our reports concerning the reaction products from calcium halides and the amino acid proline, two different solids were found for the reaction of l- and dl-proline with CaI_2_. The enanti­opure amino acid yields the one-dimensional coordination polymer *catena*-poly[[aqua-μ_3_-l-proline-tetra-μ_2_-l-proline-dicalcium] tetra­iodide 1.7-hydrate], {[Ca_2_(C_5_H_9_NO_2_)_5_(H_2_O)]I_4_·1.7H_2_O}_*n*_, (1), with two independent Ca^2+^ cations in characteristic seven- and eightfold coordination. Five symmetry-independent zwitterionic l-proline mol­ecules bridge the metal sites into a cationic polymer. Racemic proline forms with Ca^2+^ cations heterochiral chains of the one-dimensional polymer *catena*-poly[[di­aquadi-μ_2_-dl-proline-calcium] diiodide], {[Ca(C_5_H_9_NO_2_)_2_(H_2_O)_2_]I_2_}_*n*_, (2). The centrosymmetric structure is built by one Ca^2+^ cation that is bridged towards its symmetry equivalents by two zwitterionic proline mol­ecules. In both structures, the iodide ions remain non-coordinating and hydrogen bonds are formed between these counter-anions, the amino groups, coordinating and co-crystallized water mol­ecules. While the overall composition of (1) and (2) is in line with other structures from calcium halides and amino acids, the diversity of the carboxyl­ate coordination geometry is quite surprising.

## Abstract 2

Reactions of l- and dl-proline with CaI_2_ yields two crystalline products. The zwitterionic proline bridges the Ca cations with its carboxyl­ate group in different coordination modes to form one-dimensional coordination polymers.

## Chemical context   

The large field of crystal engineering benefits from the growing amount of structural data obtained by single-crystal diffraction. Amino acids are the building blocks of proteins and important mol­ecules for various applications in chemistry and life sciences. Their metal complexes have, however, been investigated less often than their availability suggests. Many of these studies address the amino acids in their deprotonated form in which it mostly acts as a *N*,*O* chelating ligand. (*e.g.* Ito *et al.*, 1971 ▸; Kato *et al.*, 2008 ▸; Magill *et al.*, 1993 ▸; Marandi & Shahbakhsh, 2007 ▸; Mathieson & Welsh, 1952 ▸; Mikhalyova *et al.*, 2010 ▸; Oki & Yoneda, 1981 ▸). In contrast, the zwitterionic overall neutral amino acids show more analogy to carboxyl­ates; for these, a large variety of coordination modes has been established (Batten *et al.*, 2008 ▸). While the protonated amino group is no longer nucleophilic, it may act as a hydrogen-bond donor. The pattern formed by these inter­actions also depends on the chirality of the enanti­opure or racemic amino acid. When both carboxyl­ate coordination and inter­molecular hydrogen bonds are taken into account, a large number of potentially competitive structures arises and subtle changes in the coordination chemistry may determine which product will be obtained. An overview of the crystal chemistry of amino acids has been published by Fleck & Petrosyan (2014 ▸). We here complete our reports concerning the reaction products from calcium halides and the amino acid proline. In this context, we encountered coordination polymers, isoreticular coordination networks, and polymorphism (Lamberts *et al.*, 2014*b* ▸; Lamberts *et al.*, 2015 ▸). The two structures reported here are coordination polymers obtained from calcium iodide and proline: the scheme shows that compounds (1) and (2) form from enanti­opure l-proline and racemic proline, respectively.
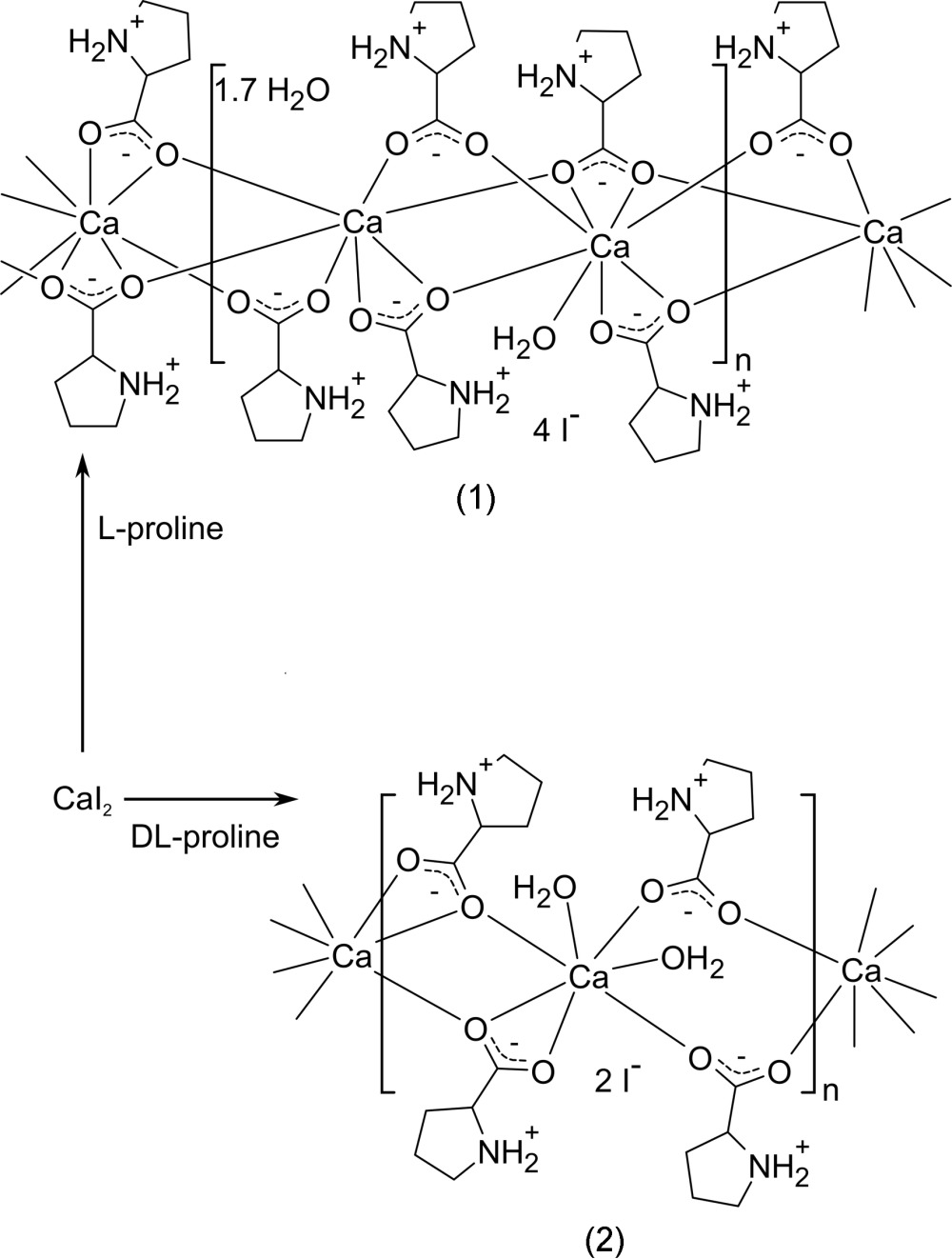


## Structural commentary   

**Figure 1:**
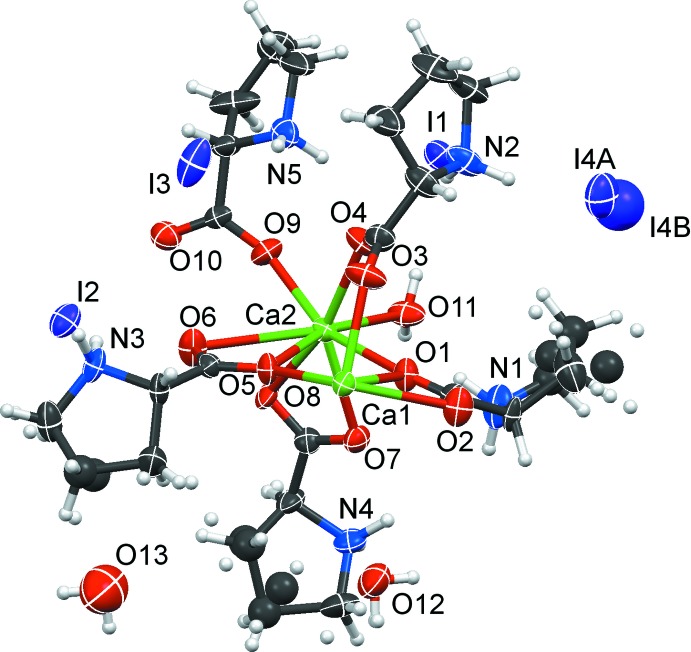
The asymmetric unit of (1). Displacement ellipsoid are shown at the 80% probability level.

Compound (1) crystallizes in the chiral ortho­rhom­bic space group *P*2_1_2_1_2_1_ with two calcium cations, five proline ligands, one coordinating water ligand, 1.7 non-coordinating water mol­ecules and four iodide anions in the asymmetric unit; all constituents are necessarily located in general positions (Fig. 1 ▸).

The five independent proline mol­ecules show three different coordination modes; in the following discussion, they are labelled according to their N atom. Proline 1 acts as a chelating ligand towards Ca1 and simultaneously as a bridge to Ca2 in a μ_2_-κ^2^:κ^1^ configuration. An analogous situation is found for proline 4, chelating Ca2 and bridging towards Ca1^iii^ [(iii) = −*x* + 1, *y* + 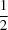, −*z* + 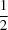]. Proline 3 connects three Ca positions in a μ_3_-κ^2^:κ^2^ coordination mode. The remaining proline ligands (2 and 5) do not chelate but only bridge two cations in a *syn–syn* configuration. Herein, proline 2 shows a more symmetric coordination, being located approximately in the middle of Ca1 and Ca2, whereas proline 5 is strongly dislocated towards Ca1.

In view of the strongly ionic nature of an inter­action between a carboxyl­ate and a calcium dication, the 3.040 (5) Å distance between Ca1 and O9^i^ [(i) = −*x* + 1, y − 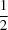, −*z* + 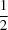] represents an additional, energetically favourable contact which, however, is much longer than a classical coordinative bond and does not affect the topology of the compound.

We mentioned in our earlier direct comparison between coordination polymers based on Ca^2+^ and Mn^2+^ (Lamberts *et al.*, 2014*a* ▸) that the absence of crystal field effects is reflected in variable and often less regular coordination spheres about the alkaline earth cation. The two cations in (1) have significantly different coordination environments: Ca1 is seven-coordin­ated by carboxyl­ato O atoms, while Ca2 offers an additional coordination site towards the water ligand to complete an eightfold coordination environment. The atoms around Ca1 are provided by two oxygen atoms of the chelating part of proline 1, and five single oxygen atoms from different bridging proline mol­ecules. Ca2 is coordinated by two chelating carboxyl­ato groups. Only three additional Ca⋯O contacts are formed from neighbouring, bridging proline ligands, whereas the remaining coordination partner is the coordinating water mol­ecule. Each Ca^2+^ cation is coordinated by the independent *syn–syn* bridging proline ligands 2 and 5; they are arranged on opposite sides around Ca1 and next to each other around Ca2.

**Figure 2:**
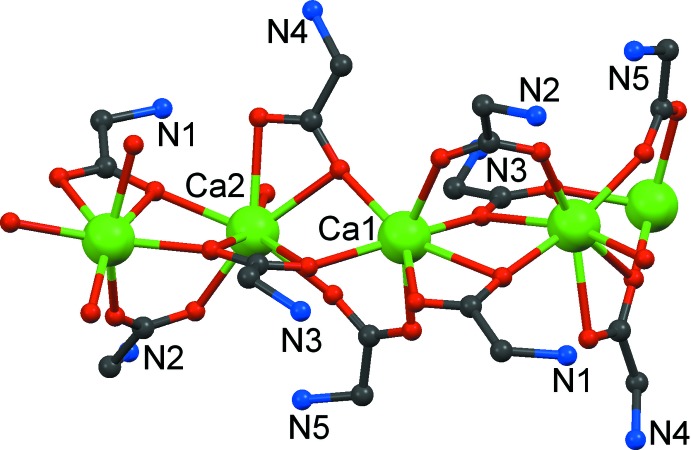
The polymeric chain of (1). H atoms and C atoms of the proline ring have been omitted for clarity.

Overall, a one dimensional coordination polymer is formed (Fig. 2 ▸). The chain extends along *b*; its projection on the *bc* plane is a sinusoidal curve, with alternating Ca1 and Ca2 positions. Each chain segment is triple bridged with two very similar independent Ca⋯Ca separations of 3.814 (2) and 3.832 (2) Å. The μ_3_-κ^2^:κ^2^ proline 3 coordinates within the sinusoidal plane in the concave parts, while proline 1 and the aqua ligand coordinate on the convex side. Selected distances are compiled in Table 1 ▸.

The iodide I4 shows positional disorder over two mutually exclusive sites, and three proline mol­ecules exhibit slight disorder of carbon atoms of the five-membered proline envelopes.

**Figure 3:**
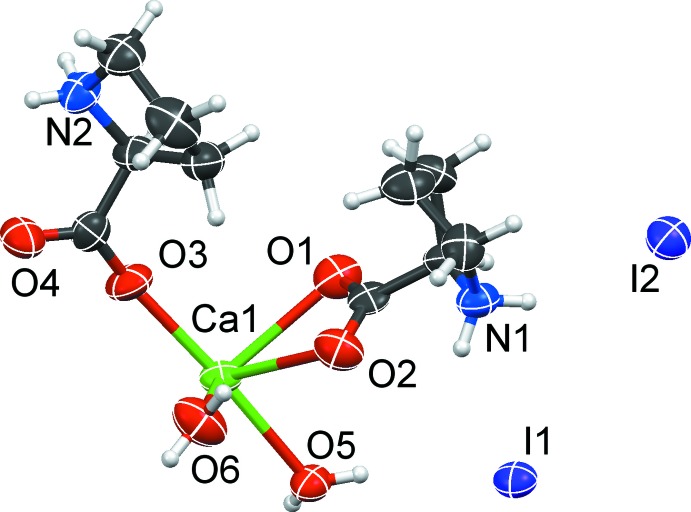
The asymmetric unit of (2). Displacement ellipsoid are shown at the 80% probability level.

Coordination polymer (2) forms under similar conditions as (1) but from racemic proline. The compound crystallizes in space group *P* 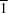 with one Ca^II^ cation, two proline ligands and two water ligands and two non-coordinating iodide anions in the asymmetric unit, all in general positions (Fig. 3 ▸).

One proline mol­ecule chelates the calcium cation with its carboxyl­ato group and additionally bridges towards a second calcium of the polymer chain (μ_2_-κ^2^:κ^1^). The other proline mol­ecule only bridges two adjacent calcium atoms in a *syn–anti* conformation (μ_2_-κ^1^:κ^1^).

**Figure 4:**
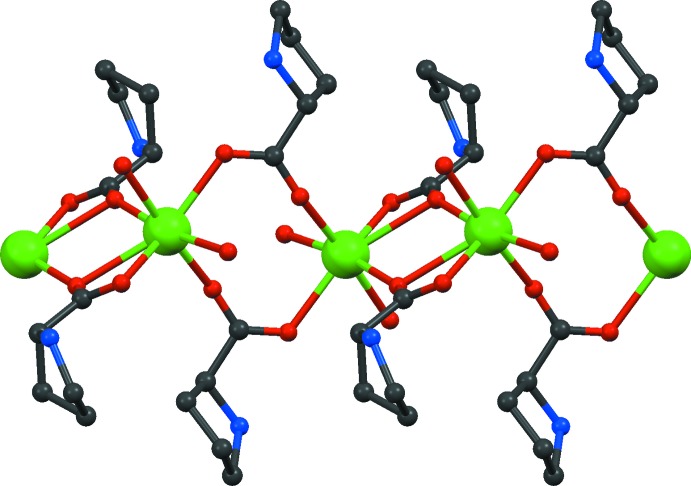
The polymeric chain of (2). H atoms have been omitted for clarity.

Together with the two aqua ligands, this results in a sevenfold coordination of the Ca^2+^ cation. Since the inversion centres lie in between the calcium atoms, two different chain connections are obtained: one is built by two simultaneously bridging and chelating proline ligands [Ca⋯Ca = 4.032 (4) Å], the other one by two *syn–anti* bridging proline ligands [Ca⋯Ca = 4.829 (4) Å, parallelogram-shaped motif]. Overall, a zigzag-shaped polymer chain is formed which extends along the shortest unit-cell axis *a* (Fig. 4 ▸). Selected distances are given in Table 2 ▸.

## Supra­molecular features   

**Figure 5:**
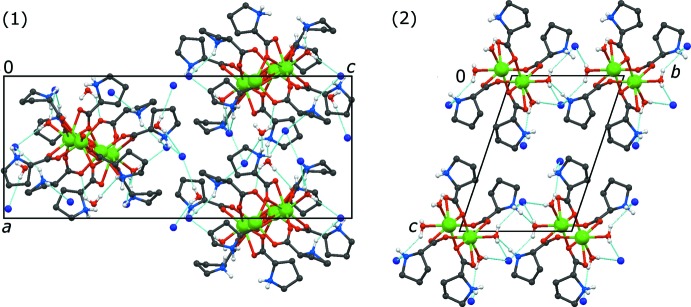
Hydrogen-bond networks formed in (1) (left) and (2) (right). Hydrogen bonds are drawn as light-blue dashed lines.

Since most hydrogen atoms in (1) have been constrained to calculated positions, their relevance should not be overestimated. The following points should, however, be mentioned: all hydrogen-bond donors find suitable acceptors. Most hydrogen bonds involve iodide and hence occur between different residues. However, only a few hydrogen bonds actually connect two neighbouring chains, resulting in an overall three-dimensional network (Fig. 5 ▸). Inter­estingly, only one of the five proline mol­ecules contributes to an N—H⋯O hydrogen bond along the chain [N3—H3*A*⋯O2^iii^; (iii) = −*x* + 1, *y* + 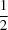, −*z* + 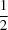].

Each of the two independent aqua ligands in (2) donates hydrogen bonds towards two iodides. The amino group associated with N2 on the one hand also forms a hydrogen bond towards iodide, on the other hand directly connects two neighbouring chains by finding a coordinating water mol­ecule as acceptor. N1 also inter­acts with an iodide counter-anion. This second NH donor can, however, not be unambiguously assigned to a hydrogen-bond acceptor: Two iodide anions are situated in its vicinity and may be regarded as acceptors for a bifurcated hydrogen bond with H⋯I distances of 3.24 (5) and 3.33 (8) Å. Overall, a two-dimensional framework is formed in the *ab* plane (Fig. 5 ▸). A complete overview of hydrogen-bond geometries is given in Tables 3 ▸ and 4 ▸.

## Database survey   

Database searches (Groom & Allen, 2014 ▸) were performed using the Cambridge Crystallographic Database (CSD, Version 5.36, including updates until November 2014). All searches were restricted to error-free entries for which 3D coordinates were available. A search for structures containing calcium and proline or derivatives in any protonation state comes up with eight hits. Six of them correspond to the aforementioned structures published by our group (Lamberts *et al.*, 2014**a* ▸,b* ▸, 2015 ▸). These are coordination polymers and networks based on calcium chloride and bromide with both l-proline and dl-proline. The other two structures are a mol­ecular complex with deprotonated *N*,*O*-chelating hy­droxy­proline (Kim *et al.*, 1985 ▸), and a coordination network of calcium pyroglutamate (Schmidbaur *et al.*, 1991 ▸).

## Synthesis and crystallization   

Single crystals of (1) were obtained by dissolving 92 mg (0.8 mmol) l-proline in 1 ml of aqueous 0.4 molar CaI_2_ solution. The solvent was evaporated under controlled conditions (Lamberts *et al.*, 2014*b* ▸) at 313 K. Suitable crystals were obtained after 5 d as yellow blocks. Crystals of (2) were obtained by using dl-proline under the same conditions and grew after 5 d as yellow plates.

## Refinement   

Crystal data, data collection and structure refinement details are summarized in Table 5 ▸. Non-hydrogen atoms were refined with anisotropic displacement parameters where possible. H atoms connected to carbon were placed in idealized positions and treated as riding, with *U*_iso_(H) = 1.2*U*_eq_(C).

In (1), significant residual density maxima indicated disorder. An alternative position for I4 was assigned and refined with an isotropic displacement parameter to a refined occupancy of 0.134 (7) (total occupancy of I4 over both positions constrained to 1). Atoms C4 and C5, C18 and C19, and C14 were also refined as split over two positions. They were given a common isotropic displacement parameter and their occupancy was refined. The occupancy of the alternative positions refined to 0.519 (12) for C4 and C5, 0.218 (12) for C18 and C19, and 0.270 (12) for C14; the occupancy sum of the alternative sites for each atom was constrained to unity. Carbon atoms connected to disordered neighbours were given two alternative geometries of calculated hydrogen positions. The occupancy of the non-coordinating water mol­ecule associated with O13 refined to 0.707 (17); tentative refinement with full occupancy resulted in an unusually large displacement parameter. Given the limited data quality, H atoms connected to nitro­gen atoms were not refined but treated as riding in idealized positions, with N—H = 0.99 Å and *U*_iso_(H) = 1.2*U*_eq_(N). The hydrogen atoms of the three water mol­ecules were modelled as oriented towards the closest acceptor and restrained to O—H distances of 0.84 Å. Further distance restraints were applied to ensure stable refinement of a reasonable hydrogen-bond geometry.

In (2), no disorder was encountered. Hydrogen atoms attached to non-carbon atoms were located in a difference Fourier map and treated as riding, with *U*_iso_(H) = 1.2*U*_eq_(non-H). N—H distances were refined with similarity restraints whereas O—H distances were restrained to 0.84 Å. H3*W* was assigned a distance restraint towards a neighbouring I1 anion to ensure suitable hydrogen-bond geometry. Reflection 0
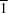
1 was omitted from the final refinement because it was obstructed by the beamstop.

## Supplementary Material

Crystal structure: contains datablock(s) global, 1, 2. DOI: 10.1107/S2056989015009597/gk2631sup1.cif

Structure factors: contains datablock(s) 1. DOI: 10.1107/S2056989015009597/gk26311sup2.hkl

Structure factors: contains datablock(s) 2. DOI: 10.1107/S2056989015009597/gk26312sup3.hkl

CCDC references: 1401789, 1401788

Additional supporting information:  crystallographic information; 3D view; checkCIF report

## Figures and Tables

**Table 1:** Selected bond lengths (Å) for (1)

Ca1—O3	2.319 (5)	Ca2—O9	2.337 (5)
Ca1—O5	2.326 (5)	Ca2—O4	2.368 (5)
Ca1—O6^i^	2.353 (5)	Ca2—O11	2.378 (5)
Ca1—O8^i^	2.358 (5)	Ca2—O1	2.378 (5)
Ca1—O10^i^	2.393 (5)	Ca2—O5	2.442 (5)
Ca1—O2	2.477 (5)	Ca2—O8	2.501 (5)
Ca1—O1	2.617 (5)	Ca2—O7	2.572 (5)
Ca1—Ca2	3.8144 (18)	Ca2—O6	2.820 (5)
Ca1—Ca2^i^	3.8315 (18)		

Symmetry code: (i) 
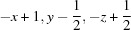
.

**Table 2:** Selected bond lengths (Å) for (2)

Ca1—O1	2.621 (6)	Ca1—O1^ii^	2.323 (5)
Ca1—O2	2.489 (6)	Ca1—O3	2.252 (5)
Ca1—O4^i^	2.396 (5)	Ca1—Ca1^i^	4.829 (4)
Ca1—O5	2.376 (5)	Ca1—Ca1^ii^	4.032 (4)
Ca1—O6	2.365 (6)		

Symmetry codes: (i) 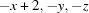; (ii) 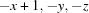.

**Table 3:** Hydrogen-bond geometry (Å, °) for (1)

*D*—H⋯*A*	*D*—H	H⋯*A*	*D*⋯*A*	*D*—H⋯*A*
N1—H1*A*⋯I3^ii^	0.99	2.60	3.526 (7)	155
N1—H1*B*⋯O11	0.99	2.10	2.996 (9)	150
N2—H2*A*⋯I1	0.99	2.75	3.603 (5)	145
N2—H2*B*⋯I4*A*	0.99	2.53	3.400 (6)	146
N3—H3*A*⋯O2^iii^	0.99	1.94	2.829 (7)	147
N3—H3*B*⋯I2	0.99	2.61	3.447 (5)	143
N4—H4*A*⋯O12	0.99	1.80	2.750 (8)	159
N4—H4*B*⋯I3^ii^	0.99	2.92	3.674 (6)	133
N5—H5*A*⋯I1	0.99	2.74	3.627 (6)	149
N5—H5*B*⋯I3	0.99	2.62	3.478 (6)	146
O11—H11*A*⋯I1	0.84 (6)	2.56 (6)	3.389 (5)	171 (7)
O11—H11*B*⋯I2^iii^	0.83 (7)	2.71 (7)	3.524 (6)	168 (5)
O12—H12*A*⋯O10^ii^	0.83 (4)	2.06 (5)	2.732 (7)	137 (4)
O12—H12*B*⋯O2^iv^	0.83 (3)	2.54 (4)	3.339 (8)	162 (7)
O13—H13*A*⋯I4*A* ^iii^	0.85 (11)	2.90 (11)	3.703 (11)	159 (10)
O13—H13*B*⋯I4*A* ^v^	0.85 (8)	2.76 (8)	3.598 (11)	172 (10)

Symmetry codes: (ii) 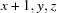; (iii) 
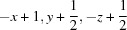
; (iv) 
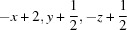
; (v) 
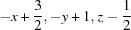
.

**Table 4:** Hydrogen-bond geometry (Å, °) for (2)

*D*—H⋯*A*	*D*—H	H⋯*A*	*D*⋯*A*	*D*—H⋯*A*
N1—H1*A*⋯I2	0.85 (7)	2.67 (7)	3.459 (8)	154 (7)
N1—H1*B*⋯I1	0.86 (7)	3.33 (7)	3.809 (7)	118 (6)
N1—H1*B*⋯I2^iii^	0.86 (7)	3.24 (7)	3.695 (7)	116 (5)
N2—H2*A*⋯I1^iv^	0.85 (6)	2.88 (6)	3.700 (8)	161 (7)
N2—H2*B*)⋯O5^v^	0.86 (6)	2.13 (7)	2.904 (9)	151 (7)
O5—H1*W*⋯I1	0.84 (7)	2.68 (7)	3.486 (6)	163 (6)
O5—H2*W*⋯I1^vi^	0.83 (3)	2.77 (6)	3.491 (6)	147 (7)
O6—H3*W*⋯I1^vii^	0.87 (6)	2.65 (6)	3.509 (7)	167 (5)
O6—H4*W*⋯I2^vii^	0.84 (5)	2.77 (4)	3.543 (6)	155 (7)

Symmetry codes: (iii) 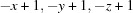; (iv) 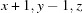; (v) 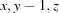; (vi) 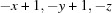; (vii) 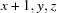.

**Table 5:** Experimental details

	(1)	(2)
Crystal data		
Chemical formula	[Ca_2_(C_5_H_9_NO_2_)_5_(H_2_O)]I_4_·1.7H_2_O	[Ca(C_5_H_9_NO_2_)_2_(H_2_O)_2_]I_2_
*M*_r_	1212.21	560.17
Crystal system, space group	Orthorhombic, *P*2_1_2_1_2_1_	Triclinic, *P* 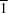
Temperature (K)	100	100
*a*, *b*, *c* (Å)	11.5276 (9), 12.7878 (10), 28.285 (2)	7.958 (7), 9.080 (8), 13.591 (11)
α, β, γ (°)	90, 90, 90	105.757 (10), 104.501 (11), 97.911 (12)
*V* (Å^3^)	4169.6 (5)	892.5 (13)
*Z*	4	2
Radiation type	Mo *K*α	Mo *K*α
μ (mm^−1^)	3.29	3.84
Crystal size (mm)	0.22 × 0.20 × 0.10	0.22 × 0.13 × 0.05

Data collection		
Diffractometer	Bruker D8 with APEX CCD area detector and Incoatec microsource	Bruker D8 with APEX CCD area detector and Incoatec microsource
Absorption correction	Multi-scan (*SADABS*; Bruker, 2008 ▸)	Multi-scan (*SADABS*; Bruker, 2008 ▸)
*T*_min_, *T*_max_	0.563, 0.746	0.447, 0.745
No. of measured, independent and observed [*I* > 2σ(*I*)] reflections	58117, 10475, 9862	8805, 3533, 2603
*R*_int_	0.053	0.079
(sin θ/λ)_max_ (Å^−1^)	0.669	0.620

Refinement		
*R*[*F* ^2^ > 2σ(*F* ^2^)], *wR*(*F* ^2^), *S*	0.036, 0.072, 1.10	0.047, 0.116, 1.02
No. of reflections	10475	3533
No. of parameters	455	214
No. of restraints	10	29
H-atom treatment	H atoms treated by a mixture of independent and constrained refinement	H atoms treated by a mixture of independent and constrained refinement
Δρ_max_, Δρ_min_ (e Å^−3^)	0.93, −0.66	1.05, −2.20
Absolute structure	Flack *x* determined using 4114 quotients [(*I* ^+^)−(*I* ^−^)]/[(*I* ^+^)+(*I* ^−^)] (Parsons & Flack, 2004 ▸)	–
Absolute structure parameter	0.023 (8)	–

Computer programs: *SMART* and *SAINT-Plus* (Bruker, 2008 ▸), *SHELXS97* (Sheldrick, 2008 ▸), *SHELXL2013* (Sheldrick, 2015 ▸) and *Mercury* (Macrae *et al.*, 2008 ▸).

## supplementary crystallographic information

### (1) catena-poly[[aqua-µ3-L-proline-tetra-µ2-L-proline-dicalcium] tetraiodide 1.7-hydrate] Crystal data

**Table:**

[Ca_2_(C_5_H_9_NO_2_)_5_(H_2_O)]I_4_·1.7H_2_O	*D*_x_ = 1.931 Mg m^−^^3^
*M**_r_* = 1212.21	Mo *K*α radiation, λ = 0.71073 Å
Orthorhombic, *P*2_1_2_1_2_1_	Cell parameters from 9906 reflections
*a* = 11.5276 (9) Å	θ = 2.4–26.3°
*b* = 12.7878 (10) Å	µ = 3.29 mm^−^^1^
*c* = 28.285 (2) Å	*T* = 100 K
*V* = 4169.6 (5) Å^3^	Block, yellow
*Z* = 4	0.22 × 0.20 × 0.10 mm
*F*(000) = 2356	

### (1) catena-poly[[aqua-µ3-L-proline-tetra-µ2-L-proline-dicalcium] tetraiodide 1.7-hydrate] Data collection

**Table:**

Bruker D8 with APEX CCD area detector and Incoatec microsource diffractometer	9862 reflections with *I* > 2σ(*I*)
ω scans	*R*_int_ = 0.053
Absorption correction: multi-scan (*SADABS*; Bruker, 2008)	θ_max_ = 28.4°, θ_min_ = 1.9°
*T*_min_ = 0.563, *T*_max_ = 0.746	*h* = −15→15
58117 measured reflections	*k* = −17→17
10475 independent reflections	*l* = −37→37

### (1) catena-poly[[aqua-µ3-L-proline-tetra-µ2-L-proline-dicalcium] tetraiodide 1.7-hydrate] Refinement

**Table:**

Refinement on *F*^2^	Hydrogen site location: mixed
Least-squares matrix: full	H atoms treated by a mixture of independent and constrained refinement
*R*[*F*^2^ > 2σ(*F*^2^)] = 0.036	*w* = 1/[σ^2^(*F*_o_^2^) + (0.005*P*)^2^ + 7.*P*] where *P* = (*F*_o_^2^ + 2*F*_c_^2^)/3
*wR*(*F*^2^) = 0.072	(Δ/σ)_max_ < 0.001
*S* = 1.10	Δρ_max_ = 0.93 e Å^−^^3^
10475 reflections	Δρ_min_ = −0.66 e Å^−^^3^
455 parameters	Absolute structure: Flack *x* determined using 4114 quotients [(*I*^+^)-(*I*^-^)]/[(*I*^+^)+(*I*^-^)] (Parsons & Flack, 2004)
10 restraints	Absolute structure parameter: 0.023 (8)

### (1) catena-poly[[aqua-µ3-L-proline-tetra-µ2-L-proline-dicalcium] tetraiodide 1.7-hydrate] Special details

**Table:**

Geometry. All e.s.d.'s (except the e.s.d. in the dihedral angle between two l.s. planes) are estimated using the full covariance matrix. The cell e.s.d.'s are taken into account individually in the estimation of e.s.d.'s in distances, angles and torsion angles; correlations between e.s.d.'s in cell parameters are only used when they are defined by crystal symmetry. An approximate (isotropic) treatment of cell e.s.d.'s is used for estimating e.s.d.'s involving l.s. planes.

### (1) catena-poly[[aqua-µ3-L-proline-tetra-µ2-L-proline-dicalcium] tetraiodide 1.7-hydrate] Fractional atomic coordinates and isotropic or equivalent isotropic displacement parameters (Å^2^)

**Table:**

	*x*	*y*	*z*	*U*_iso_*/*U*_eq_	Occ. (<1)
I1	0.50063 (4)	0.78534 (4)	0.46773 (2)	0.02285 (11)	
I2	0.09876 (4)	0.41042 (4)	0.14734 (2)	0.02416 (11)	
I3	0.11459 (5)	0.59585 (4)	0.30876 (2)	0.03516 (14)	
I4A	0.56556 (15)	0.30647 (9)	0.51264 (3)	0.0380 (4)	0.866 (7)
I4B	0.6104 (12)	0.3290 (8)	0.5192 (3)	0.063 (3)*	0.134 (7)
Ca1	0.53455 (11)	0.37203 (10)	0.27820 (5)	0.0131 (3)	
Ca2	0.55317 (12)	0.66087 (10)	0.31101 (5)	0.0138 (3)	
O1	0.6580 (4)	0.5048 (4)	0.32742 (16)	0.0182 (10)	
O2	0.7001 (5)	0.3367 (4)	0.33170 (18)	0.0239 (12)	
O3	0.4226 (4)	0.4116 (4)	0.34390 (16)	0.0247 (11)	
O4	0.4391 (4)	0.5772 (4)	0.36946 (16)	0.0236 (11)	
O5	0.4838 (4)	0.5378 (4)	0.25134 (16)	0.0183 (10)	
O6	0.4223 (5)	0.6927 (4)	0.22803 (16)	0.0223 (11)	
O7	0.7622 (4)	0.6802 (4)	0.28044 (16)	0.0183 (10)	
O8	0.6321 (4)	0.7962 (4)	0.25585 (16)	0.0204 (10)	
O9	0.4242 (4)	0.7994 (4)	0.32275 (16)	0.0213 (11)	
O10	0.3145 (4)	0.9020 (4)	0.27825 (16)	0.0216 (10)	
O11	0.6681 (5)	0.7372 (4)	0.37170 (18)	0.0226 (11)	
H11B	0.716 (6)	0.783 (5)	0.364 (3)	0.027*	
H11A	0.632 (6)	0.755 (6)	0.3963 (18)	0.027*	
O12	1.0987 (5)	0.9338 (4)	0.24099 (19)	0.0306 (13)	
H12A	1.141 (4)	0.928 (7)	0.2647 (10)	0.037*	
H12B	1.154 (3)	0.924 (6)	0.2225 (8)	0.037*	
O13	0.6478 (10)	0.6644 (9)	0.0612 (4)	0.059 (4)	0.707 (17)
H13B	0.712 (6)	0.670 (13)	0.047 (4)	0.070*	0.707 (17)
H13A	0.612 (10)	0.691 (12)	0.038 (3)	0.070*	0.707 (17)
N1	0.8513 (6)	0.5710 (5)	0.3712 (2)	0.0291 (15)	
H1A	0.9093	0.5919	0.3472	0.035*	
H1B	0.7779	0.6077	0.3639	0.035*	
N2	0.4256 (6)	0.5121 (4)	0.4621 (2)	0.0242 (14)	
H2A	0.4539	0.5790	0.4488	0.029*	
H2B	0.4922	0.4751	0.4765	0.029*	
N3	0.2892 (5)	0.6171 (4)	0.1571 (2)	0.0192 (13)	
H3A	0.2875	0.6855	0.1735	0.023*	
H3B	0.2114	0.5849	0.1598	0.023*	
N4	0.9412 (5)	0.7729 (5)	0.2348 (2)	0.0218 (13)	
H4A	0.9849	0.8373	0.2425	0.026*	
H4B	0.9402	0.7276	0.2632	0.026*	
N5	0.2588 (5)	0.7619 (5)	0.3864 (2)	0.0222 (14)	
H5A	0.3409	0.7607	0.3967	0.027*	
H5B	0.2396	0.6934	0.3721	0.027*	
C1	0.7208 (6)	0.4304 (5)	0.3402 (2)	0.0171 (14)	
C2	0.8318 (6)	0.4551 (5)	0.3680 (2)	0.0174 (14)	
H2	0.8996	0.4219	0.3519	0.021*	
C3A	0.8275 (8)	0.4183 (7)	0.4196 (3)	0.035 (2)	0.481 (12)
H3A1	0.8986	0.3795	0.4285	0.042*	0.481 (12)
H3A2	0.7586	0.3742	0.4258	0.042*	0.481 (12)
C4A	0.8195 (15)	0.5273 (13)	0.4458 (6)	0.0263 (10)*	0.481 (12)
H4A1	0.7378	0.5509	0.4479	0.032*	0.481 (12)
H4A2	0.8516	0.5221	0.4782	0.032*	0.481 (12)
C5A	0.8904 (16)	0.6024 (14)	0.4161 (6)	0.0263 (10)*	0.481 (12)
H5A1	0.8710	0.6763	0.4229	0.032*	0.481 (12)
H5A2	0.9748	0.5912	0.4201	0.032*	0.481 (12)
C3B	0.8275 (8)	0.4183 (7)	0.4196 (3)	0.035 (2)	0.519 (12)
H3B1	0.7466	0.4191	0.4313	0.042*	0.519 (12)
H3B2	0.8577	0.3460	0.4221	0.042*	0.519 (12)
C4B	0.8989 (14)	0.4893 (11)	0.4475 (5)	0.0263 (10)*	0.519 (12)
H4B1	0.9827	0.4784	0.4418	0.032*	0.519 (12)
H4B2	0.8823	0.4838	0.4817	0.032*	0.519 (12)
C5B	0.8564 (15)	0.5922 (14)	0.4269 (5)	0.0263 (10)*	0.519 (12)
H5B1	0.9110	0.6497	0.4343	0.032*	0.519 (12)
H5B2	0.7788	0.6103	0.4394	0.032*	0.519 (12)
C6	0.4157 (6)	0.4818 (5)	0.3747 (2)	0.0199 (15)	
C7	0.3731 (7)	0.4458 (6)	0.4233 (2)	0.0235 (16)	
H7	0.3953	0.3710	0.4283	0.028*	
C8	0.2421 (7)	0.4584 (7)	0.4310 (3)	0.0323 (19)	
H8A	0.1990	0.3970	0.4188	0.039*	
H8B	0.2126	0.5224	0.4154	0.039*	
C9	0.2318 (8)	0.4665 (7)	0.4850 (3)	0.035 (2)	
H9A	0.1578	0.5001	0.4942	0.042*	
H9B	0.2362	0.3965	0.4999	0.042*	
C10	0.3337 (8)	0.5329 (6)	0.4991 (3)	0.0308 (19)	
H10A	0.3620	0.5130	0.5309	0.037*	
H10B	0.3121	0.6078	0.4995	0.037*	
C11	0.4304 (6)	0.5976 (5)	0.2230 (2)	0.0145 (13)	
C12	0.3783 (6)	0.5474 (5)	0.1796 (2)	0.0174 (14)	
H12	0.3422	0.4790	0.1883	0.021*	
C13A	0.4678 (7)	0.5310 (7)	0.1402 (3)	0.0322 (19)	0.730 (12)
H13C	0.5022	0.4601	0.1418	0.039*	0.730 (12)
H13D	0.5305	0.5837	0.1419	0.039*	0.730 (12)
C14A	0.3950 (10)	0.5447 (8)	0.0946 (4)	0.0263 (10)*	0.730 (12)
H14A	0.4451	0.5608	0.0672	0.032*	0.730 (12)
H14B	0.3492	0.4810	0.0877	0.032*	0.730 (12)
C15A	0.3196 (8)	0.6325 (6)	0.1059 (3)	0.0274 (18)	0.730 (12)
H15A	0.2491	0.6316	0.0859	0.033*	0.730 (12)
H15B	0.3605	0.6998	0.1011	0.033*	0.730 (12)
C13B	0.4678 (7)	0.5310 (7)	0.1402 (3)	0.0322 (19)	0.270 (12)
H13E	0.4528	0.4647	0.1231	0.039*	0.270 (12)
H13F	0.5474	0.5290	0.1533	0.039*	0.270 (12)
C14B	0.452 (3)	0.627 (2)	0.1061 (10)	0.0263 (10)*	0.270 (12)
H14C	0.4877	0.6916	0.1193	0.032*	0.270 (12)
H14D	0.4844	0.6134	0.0743	0.032*	0.270 (12)
C15B	0.3196 (8)	0.6325 (6)	0.1059 (3)	0.0274 (18)	0.270 (12)
H15C	0.2921	0.7012	0.0943	0.033*	0.270 (12)
H15D	0.2860	0.5765	0.0860	0.033*	0.270 (12)
C16	0.7340 (6)	0.7567 (5)	0.2558 (2)	0.0162 (14)	
C17A	0.8181 (6)	0.8005 (6)	0.2205 (2)	0.0214 (15)	0.782 (12)
H17A	0.8082	0.8777	0.2167	0.026*	0.782 (12)
C18A	0.8031 (10)	0.7417 (9)	0.1718 (4)	0.0263 (10)*	0.782 (12)
H18A	0.7791	0.6682	0.1766	0.032*	0.782 (12)
H18B	0.7451	0.7772	0.1515	0.032*	0.782 (12)
C19A	0.9234 (8)	0.7480 (8)	0.1502 (3)	0.0263 (10)*	0.782 (12)
H19A	0.9417	0.8193	0.1389	0.032*	0.782 (12)
H19B	0.9335	0.6974	0.1241	0.032*	0.782 (12)
C20A	0.9976 (7)	0.7173 (7)	0.1949 (3)	0.0341 (18)	0.782 (12)
H20A	0.9963	0.6407	0.2000	0.041*	0.782 (12)
H20B	1.0791	0.7403	0.1911	0.041*	0.782 (12)
C17B	0.8181 (6)	0.8005 (6)	0.2205 (2)	0.0214 (15)	0.218 (12)
H17B	0.8127	0.8781	0.2242	0.026*	0.218 (12)
C18B	0.812 (3)	0.784 (3)	0.1740 (13)	0.0263 (10)*	0.218 (12)
H18C	0.8451	0.8431	0.1561	0.032*	0.218 (12)
H18D	0.7305	0.7729	0.1639	0.032*	0.218 (12)
C19B	0.884 (3)	0.684 (3)	0.1670 (12)	0.0263 (10)*	0.218 (12)
H19C	0.9006	0.6698	0.1332	0.032*	0.218 (12)
H19D	0.8468	0.6217	0.1814	0.032*	0.218 (12)
C20B	0.9976 (7)	0.7173 (7)	0.1949 (3)	0.0341 (18)	0.218 (12)
H20C	1.0432	0.6561	0.2057	0.041*	0.218 (12)
H20D	1.0477	0.7643	0.1760	0.041*	0.218 (12)
C21	0.3319 (6)	0.8491 (5)	0.3143 (2)	0.0142 (13)	
C22	0.2389 (6)	0.8479 (5)	0.3520 (2)	0.0178 (14)	
H22	0.1625	0.8356	0.3362	0.021*	
C23	0.2274 (7)	0.9435 (6)	0.3836 (3)	0.033 (2)	
H23A	0.1725	0.9946	0.3698	0.040*	
H23B	0.3036	0.9780	0.3880	0.040*	
C24	0.1817 (8)	0.9015 (9)	0.4305 (3)	0.046 (3)	
H24A	0.2325	0.9242	0.4568	0.055*	
H24B	0.1024	0.9282	0.4365	0.055*	
C25	0.1804 (7)	0.7848 (8)	0.4272 (3)	0.039 (2)	
H25A	0.2100	0.7526	0.4566	0.047*	
H25B	0.1010	0.7586	0.4211	0.047*	

### (1) catena-poly[[aqua-µ3-L-proline-tetra-µ2-L-proline-dicalcium] tetraiodide 1.7-hydrate] Atomic displacement parameters (Å^2^)

**Table:**

	*U*^11^	*U*^22^	*U*^33^	*U*^12^	*U*^13^	*U*^23^
I1	0.0253 (2)	0.0261 (2)	0.01717 (19)	0.0004 (2)	−0.0012 (2)	−0.00403 (18)
I2	0.0162 (2)	0.0236 (2)	0.0327 (2)	−0.0018 (2)	−0.0035 (2)	0.0007 (2)
I3	0.0306 (3)	0.0202 (2)	0.0547 (3)	0.0013 (2)	−0.0197 (3)	−0.0077 (2)
I4A	0.0449 (7)	0.0271 (5)	0.0421 (4)	0.0087 (4)	−0.0073 (4)	0.0069 (3)
Ca1	0.0131 (7)	0.0115 (6)	0.0148 (6)	−0.0007 (5)	−0.0016 (5)	−0.0011 (5)
Ca2	0.0152 (7)	0.0120 (6)	0.0143 (6)	−0.0013 (5)	0.0007 (5)	0.0003 (5)
O1	0.020 (3)	0.014 (2)	0.021 (2)	−0.003 (2)	−0.006 (2)	0.0020 (19)
O2	0.028 (3)	0.013 (2)	0.030 (3)	−0.003 (2)	−0.009 (2)	−0.002 (2)
O3	0.031 (3)	0.024 (3)	0.020 (2)	−0.010 (2)	0.010 (2)	−0.008 (2)
O4	0.032 (3)	0.023 (3)	0.016 (2)	−0.005 (2)	0.005 (2)	0.001 (2)
O5	0.019 (3)	0.019 (2)	0.016 (2)	0.000 (2)	−0.007 (2)	0.0007 (18)
O6	0.031 (3)	0.011 (2)	0.025 (3)	0.001 (2)	−0.009 (2)	−0.0040 (19)
O7	0.017 (3)	0.017 (2)	0.022 (2)	0.003 (2)	0.001 (2)	0.0052 (19)
O8	0.009 (2)	0.028 (3)	0.024 (2)	0.002 (2)	−0.0005 (19)	0.010 (2)
O9	0.014 (3)	0.022 (3)	0.028 (3)	0.003 (2)	0.002 (2)	−0.001 (2)
O10	0.021 (3)	0.023 (3)	0.020 (2)	−0.004 (2)	0.002 (2)	0.001 (2)
O11	0.018 (3)	0.026 (3)	0.024 (3)	−0.009 (2)	0.007 (2)	−0.005 (2)
O12	0.020 (3)	0.035 (3)	0.036 (3)	0.002 (3)	−0.002 (2)	0.005 (3)
O13	0.054 (7)	0.055 (7)	0.067 (8)	0.001 (6)	0.007 (6)	0.022 (6)
N1	0.029 (4)	0.022 (4)	0.036 (4)	−0.007 (3)	−0.013 (3)	0.000 (3)
N2	0.040 (4)	0.015 (3)	0.018 (3)	0.000 (3)	0.007 (3)	−0.002 (2)
N3	0.015 (3)	0.016 (3)	0.026 (3)	0.000 (2)	−0.010 (2)	−0.001 (2)
N4	0.014 (3)	0.027 (3)	0.025 (3)	−0.002 (3)	0.005 (2)	0.001 (3)
N5	0.019 (3)	0.029 (4)	0.019 (3)	−0.004 (3)	−0.002 (2)	0.001 (3)
C1	0.017 (3)	0.017 (4)	0.017 (3)	−0.003 (3)	−0.001 (3)	−0.002 (3)
C2	0.019 (4)	0.010 (3)	0.023 (3)	0.005 (3)	−0.010 (3)	0.000 (3)
C3A	0.042 (5)	0.039 (5)	0.026 (4)	−0.009 (4)	−0.009 (4)	0.007 (4)
C3B	0.042 (5)	0.039 (5)	0.026 (4)	−0.009 (4)	−0.009 (4)	0.007 (4)
C6	0.021 (4)	0.020 (3)	0.018 (3)	−0.001 (3)	0.001 (3)	−0.003 (3)
C7	0.034 (5)	0.021 (4)	0.016 (3)	−0.008 (3)	0.002 (3)	−0.004 (3)
C8	0.028 (5)	0.047 (5)	0.023 (4)	−0.009 (4)	0.006 (3)	0.001 (4)
C9	0.041 (5)	0.039 (5)	0.026 (4)	−0.014 (4)	0.017 (4)	0.003 (4)
C10	0.045 (5)	0.031 (4)	0.016 (4)	0.001 (4)	0.011 (3)	−0.003 (3)
C11	0.015 (3)	0.014 (3)	0.014 (3)	0.000 (3)	0.001 (2)	0.003 (3)
C12	0.022 (4)	0.013 (3)	0.017 (3)	0.003 (3)	−0.008 (3)	0.001 (2)
C13A	0.029 (5)	0.046 (5)	0.021 (4)	0.015 (4)	−0.002 (3)	−0.002 (3)
C15A	0.039 (5)	0.020 (4)	0.023 (4)	0.004 (3)	−0.011 (3)	0.001 (3)
C13B	0.029 (5)	0.046 (5)	0.021 (4)	0.015 (4)	−0.002 (3)	−0.002 (3)
C15B	0.039 (5)	0.020 (4)	0.023 (4)	0.004 (3)	−0.011 (3)	0.001 (3)
C16	0.015 (4)	0.016 (3)	0.018 (3)	−0.003 (3)	0.000 (3)	0.001 (3)
C17A	0.012 (3)	0.025 (4)	0.027 (4)	0.003 (3)	0.005 (3)	0.010 (3)
C20A	0.022 (4)	0.046 (5)	0.034 (4)	0.010 (4)	0.010 (4)	−0.004 (4)
C17B	0.012 (3)	0.025 (4)	0.027 (4)	0.003 (3)	0.005 (3)	0.010 (3)
C20B	0.022 (4)	0.046 (5)	0.034 (4)	0.010 (4)	0.010 (4)	−0.004 (4)
C21	0.012 (3)	0.013 (3)	0.018 (3)	−0.002 (3)	0.000 (3)	−0.006 (3)
C22	0.016 (3)	0.018 (3)	0.020 (3)	0.004 (3)	0.006 (3)	0.001 (3)
C23	0.024 (4)	0.028 (4)	0.047 (5)	−0.006 (3)	0.018 (4)	−0.015 (4)
C24	0.036 (5)	0.077 (7)	0.024 (4)	0.021 (5)	−0.004 (4)	−0.024 (5)
C25	0.022 (4)	0.076 (7)	0.019 (4)	−0.005 (5)	0.008 (3)	−0.001 (4)

### (1) catena-poly[[aqua-µ3-L-proline-tetra-µ2-L-proline-dicalcium] tetraiodide 1.7-hydrate] Geometric parameters (Å, º)

**Table:**

Ca1—O3	2.319 (5)	C3A—H3A1	0.9900
Ca1—O5	2.326 (5)	C3A—H3A2	0.9900
Ca1—O6^i^	2.353 (5)	C4A—C5A	1.51 (2)
Ca1—O8^i^	2.358 (5)	C4A—H4A1	0.9900
Ca1—O10^i^	2.393 (5)	C4A—H4A2	0.9900
Ca1—O2	2.477 (5)	C5A—H5A1	0.9900
Ca1—O1	2.617 (5)	C5A—H5A2	0.9900
Ca1—Ca2	3.8144 (18)	C4B—C5B	1.52 (2)
Ca1—Ca2^i^	3.8315 (18)	C4B—H4B1	0.9900
Ca2—O9	2.337 (5)	C4B—H4B2	0.9900
Ca2—O4	2.368 (5)	C5B—H5B1	0.9900
Ca2—O11	2.378 (5)	C5B—H5B2	0.9900
Ca2—O1	2.378 (5)	C6—C7	1.531 (9)
Ca2—O5	2.442 (5)	C7—C8	1.534 (11)
Ca2—O8	2.501 (5)	C7—H7	1.0000
Ca2—O7	2.572 (5)	C8—C9	1.536 (11)
Ca2—O6	2.820 (5)	C8—H8A	0.9900
Ca1—C1	2.871 (7)	C8—H8B	0.9900
Ca1—C21^i^	3.049 (7)	C9—C10	1.504 (12)
Ca2—C11	2.976 (6)	C9—H9A	0.9900
O1—C1	1.249 (8)	C9—H9B	0.9900
O2—C1	1.245 (8)	C10—H10A	0.9900
O3—C6	1.254 (8)	C10—H10B	0.9900
O4—C6	1.258 (8)	C11—C12	1.510 (9)
O5—C11	1.267 (8)	C12—C13A	1.534 (10)
O6—C11	1.229 (8)	C12—H12	1.0000
O6—Ca1^ii^	2.353 (5)	C13A—C14A	1.547 (13)
O7—C16	1.245 (8)	C13A—H13C	0.9900
O8—C16	1.278 (8)	C13A—H13D	0.9900
O8—Ca1^ii^	2.358 (5)	C14A—C15A	1.455 (12)
O9—C21	1.262 (8)	C14A—H14A	0.9900
O9—Ca1^ii^	3.040 (5)	C14A—H14B	0.9900
O10—C21	1.240 (8)	C15A—H15A	0.9900
O10—Ca1^ii^	2.393 (5)	C15A—H15B	0.9900
O11—H11B	0.83 (3)	C14B—H14C	0.9900
O11—H11A	0.84 (3)	C14B—H14D	0.9900
O12—H12A	0.84 (3)	C16—C17A	1.501 (9)
O12—H12B	0.84 (2)	C17A—C18A	1.579 (13)
O13—H13B	0.84 (3)	C17A—H17A	1.0000
O13—H13A	0.84 (3)	C18A—C19A	1.517 (15)
N1—C5A	1.406 (18)	C18A—H18A	0.9900
N1—C2	1.502 (9)	C18A—H18B	0.9900
N1—C5B	1.599 (17)	C19A—C20A	1.575 (12)
N1—H1A	0.9900	C19A—H19A	0.9900
N1—H1B	0.9900	C19A—H19B	0.9900
N2—C7	1.511 (9)	C20A—H20A	0.9900
N2—C10	1.514 (9)	C20A—H20B	0.9900
N2—H2A	0.9900	C18B—C19B	1.54 (5)
N2—H2B	0.9900	C18B—H18C	0.9900
N3—C12	1.501 (8)	C18B—H18D	0.9900
N3—C15A	1.505 (9)	C19B—H19C	0.9900
N3—H3A	0.9900	C19B—H19D	0.9900
N3—H3B	0.9900	C21—C22	1.512 (9)
N4—C20A	1.485 (9)	C21—Ca1^ii^	3.049 (7)
N4—C17A	1.517 (9)	C22—C23	1.522 (10)
N4—H4A	0.9900	C22—H22	1.0000
N4—H4B	0.9900	C23—C24	1.526 (12)
N5—C22	1.487 (9)	C23—H23A	0.9900
N5—C25	1.495 (9)	C23—H23B	0.9900
N5—H5A	0.9900	C24—C25	1.496 (14)
N5—H5B	0.9900	C24—H24A	0.9900
C1—C2	1.535 (9)	C24—H24B	0.9900
C2—C3A	1.533 (10)	C25—H25A	0.9900
C2—H2	1.0000	C25—H25B	0.9900
C3A—C4A	1.582 (18)		
			
O3—Ca1—O5	85.58 (18)	C20A—N4—C17A	108.5 (6)
O3—Ca1—O6^i^	112.99 (18)	C20A—N4—H4A	110.0
O5—Ca1—O6^i^	156.43 (17)	C17A—N4—H4A	110.0
O3—Ca1—O8^i^	87.92 (17)	C20A—N4—H4B	110.0
O5—Ca1—O8^i^	92.09 (18)	C17A—N4—H4B	110.0
O6^i^—Ca1—O8^i^	74.98 (18)	H4A—N4—H4B	108.4
O3—Ca1—O10^i^	154.78 (18)	C22—N5—C25	105.5 (6)
O5—Ca1—O10^i^	79.57 (18)	C22—N5—H5A	110.6
O6^i^—Ca1—O10^i^	87.28 (18)	C25—N5—H5A	110.6
O8^i^—Ca1—O10^i^	112.72 (17)	C22—N5—H5B	110.6
O3—Ca1—O2	88.79 (18)	C25—N5—H5B	110.6
O5—Ca1—O2	124.01 (17)	H5A—N5—H5B	108.8
O6^i^—Ca1—O2	72.86 (17)	O2—C1—O1	124.5 (6)
O8^i^—Ca1—O2	143.35 (18)	O2—C1—C2	117.2 (6)
O10^i^—Ca1—O2	82.90 (18)	O1—C1—C2	118.3 (6)
O3—Ca1—O1	74.61 (17)	O2—C1—Ca1	59.2 (4)
O5—Ca1—O1	73.69 (16)	O1—C1—Ca1	65.7 (4)
O6^i^—Ca1—O1	123.88 (17)	C2—C1—Ca1	171.9 (5)
O8^i^—Ca1—O1	158.03 (17)	N1—C2—C3A	104.6 (6)
O10^i^—Ca1—O1	81.68 (17)	N1—C2—C1	111.0 (5)
O2—Ca1—O1	51.27 (15)	C3A—C2—C1	113.4 (6)
O3—Ca1—C1	82.51 (19)	N1—C2—H2	109.2
O5—Ca1—C1	98.66 (18)	C3A—C2—H2	109.2
O6^i^—Ca1—C1	98.12 (19)	C1—C2—H2	109.2
O8^i^—Ca1—C1	164.98 (19)	C2—C3A—C4A	100.2 (8)
O10^i^—Ca1—C1	79.75 (18)	C2—C3A—H3A1	111.7
O2—Ca1—C1	25.59 (17)	C4A—C3A—H3A1	111.7
O1—Ca1—C1	25.78 (16)	C2—C3A—H3A2	111.7
O3—Ca1—O9^i^	155.05 (17)	C4A—C3A—H3A2	111.7
O5—Ca1—O9^i^	90.62 (15)	H3A1—C3A—H3A2	109.5
O6^i^—Ca1—O9^i^	66.36 (15)	C5A—C4A—C3A	105.5 (12)
O8^i^—Ca1—O9^i^	67.56 (14)	C5A—C4A—H4A1	110.6
O10^i^—Ca1—O9^i^	46.25 (15)	C3A—C4A—H4A1	110.6
O2—Ca1—O9^i^	113.43 (16)	C5A—C4A—H4A2	110.6
O1—Ca1—O9^i^	127.79 (15)	C3A—C4A—H4A2	110.6
C1—Ca1—O9^i^	122.43 (17)	H4A1—C4A—H4A2	108.8
O3—Ca1—C21^i^	171.73 (18)	N1—C5A—C4A	98.5 (13)
O5—Ca1—C21^i^	86.23 (17)	N1—C5A—H5A1	112.1
O6^i^—Ca1—C21^i^	74.66 (17)	C4A—C5A—H5A1	112.1
O8^i^—Ca1—C21^i^	91.22 (17)	N1—C5A—H5A2	112.1
O10^i^—Ca1—C21^i^	22.46 (17)	C4A—C5A—H5A2	112.1
O2—Ca1—C21^i^	96.76 (18)	H5A1—C5A—H5A2	109.7
O1—Ca1—C21^i^	104.14 (17)	C5B—C4B—H4B1	112.0
C1—Ca1—C21^i^	99.89 (19)	C5B—C4B—H4B2	112.0
O9^i^—Ca1—C21^i^	23.91 (15)	H4B1—C4B—H4B2	109.7
O3—Ca1—Ca2	67.96 (13)	C4B—C5B—N1	104.0 (12)
O5—Ca1—Ca2	37.94 (11)	C4B—C5B—H5B1	110.9
O6^i^—Ca1—Ca2	161.81 (13)	N1—C5B—H5B1	110.9
O8^i^—Ca1—Ca2	122.92 (13)	C4B—C5B—H5B2	110.9
O10^i^—Ca1—Ca2	88.06 (13)	N1—C5B—H5B2	110.9
O2—Ca1—Ca2	89.12 (12)	H5B1—C5B—H5B2	109.0
O1—Ca1—Ca2	37.96 (11)	O3—C6—O4	126.7 (6)
C1—Ca1—Ca2	63.74 (14)	O3—C6—C7	115.4 (6)
O9^i^—Ca1—Ca2	121.02 (10)	O4—C6—C7	117.9 (6)
C21^i^—Ca1—Ca2	105.87 (13)	N2—C7—C6	110.7 (6)
O3—Ca1—Ca2^i^	122.34 (13)	N2—C7—C8	103.5 (6)
O5—Ca1—Ca2^i^	111.14 (12)	C6—C7—C8	114.3 (6)
O6^i^—Ca1—Ca2^i^	47.13 (12)	N2—C7—H7	109.4
O8^i^—Ca1—Ca2^i^	39.30 (12)	C6—C7—H7	109.4
O10^i^—Ca1—Ca2^i^	82.27 (13)	C8—C7—H7	109.4
O2—Ca1—Ca2^i^	118.50 (12)	C7—C8—C9	102.9 (7)
O1—Ca1—Ca2^i^	162.03 (12)	C7—C8—H8A	111.2
C1—Ca1—Ca2^i^	141.53 (14)	C9—C8—H8A	111.2
O9^i^—Ca1—Ca2^i^	37.58 (9)	C7—C8—H8B	111.2
C21^i^—Ca1—Ca2^i^	60.03 (13)	C9—C8—H8B	111.2
Ca2—Ca1—Ca2^i^	149.04 (4)	H8A—C8—H8B	109.1
O9—Ca2—O4	83.70 (18)	C10—C9—C8	104.0 (6)
O9—Ca2—O11	86.60 (18)	C10—C9—H9A	111.0
O4—Ca2—O11	89.47 (17)	C8—C9—H9A	111.0
O9—Ca2—O1	158.70 (18)	C10—C9—H9B	111.0
O4—Ca2—O1	76.51 (17)	C8—C9—H9B	111.0
O11—Ca2—O1	85.43 (18)	H9A—C9—H9B	109.0
O9—Ca2—O5	112.25 (17)	C9—C10—N2	105.3 (6)
O4—Ca2—O5	90.55 (16)	C9—C10—H10A	110.7
O11—Ca2—O5	161.04 (19)	N2—C10—H10A	110.7
O1—Ca2—O5	76.15 (16)	C9—C10—H10B	110.7
O9—Ca2—O8	78.20 (17)	N2—C10—H10B	110.7
O4—Ca2—O8	161.84 (18)	H10A—C10—H10B	108.8
O11—Ca2—O8	87.91 (17)	O6—C11—O5	124.1 (6)
O1—Ca2—O8	121.14 (17)	O6—C11—C12	119.0 (6)
O5—Ca2—O8	97.70 (16)	O5—C11—C12	116.8 (6)
O9—Ca2—O7	124.82 (17)	O6—C11—Ca2	70.7 (4)
O4—Ca2—O7	142.96 (17)	O5—C11—Ca2	53.4 (3)
O11—Ca2—O7	71.41 (17)	C12—C11—Ca2	170.1 (5)
O1—Ca2—O7	70.74 (16)	N3—C12—C11	111.4 (5)
O5—Ca2—O7	97.83 (17)	N3—C12—C13A	103.5 (5)
O8—Ca2—O7	51.90 (15)	C11—C12—C13A	112.5 (6)
O9—Ca2—O6	70.63 (15)	N3—C12—H12	109.8
O4—Ca2—O6	110.47 (17)	C11—C12—H12	109.8
O11—Ca2—O6	147.10 (17)	C13A—C12—H12	109.8
O1—Ca2—O6	123.75 (15)	C12—C13A—C14A	103.0 (7)
O5—Ca2—O6	48.91 (14)	C12—C13A—H13C	111.2
O8—Ca2—O6	64.88 (15)	C14A—C13A—H13C	111.2
O7—Ca2—O6	101.97 (16)	C12—C13A—H13D	111.2
O9—Ca2—C16	102.41 (19)	C14A—C13A—H13D	111.2
O4—Ca2—C16	166.73 (19)	H13C—C13A—H13D	109.1
O11—Ca2—C16	79.26 (19)	C15A—C14A—C13A	103.3 (7)
O1—Ca2—C16	95.45 (19)	C15A—C14A—H14A	111.1
O5—Ca2—C16	97.83 (18)	C13A—C14A—H14A	111.1
O8—Ca2—C16	26.30 (17)	C15A—C14A—H14B	111.1
O7—Ca2—C16	25.62 (16)	C13A—C14A—H14B	111.1
O6—Ca2—C16	82.76 (17)	H14A—C14A—H14B	109.1
O9—Ca2—C11	91.29 (18)	C14A—C15A—N3	104.4 (6)
O4—Ca2—C11	101.37 (18)	C14A—C15A—H15A	110.9
O11—Ca2—C11	168.68 (18)	N3—C15A—H15A	110.9
O1—Ca2—C11	100.18 (18)	C14A—C15A—H15B	110.9
O5—Ca2—C11	24.62 (17)	N3—C15A—H15B	110.9
O8—Ca2—C11	80.77 (17)	H15A—C15A—H15B	108.9
O7—Ca2—C11	100.99 (16)	H14C—C14B—H14D	109.7
O6—Ca2—C11	24.29 (15)	O7—C16—O8	123.4 (6)
C16—Ca2—C11	90.36 (18)	O7—C16—C17A	119.8 (6)
O9—Ca2—Ca1	137.14 (13)	O8—C16—C17A	116.6 (6)
O4—Ca2—Ca1	72.60 (12)	O7—C16—Ca2	63.3 (4)
O11—Ca2—Ca1	127.19 (14)	O8—C16—Ca2	60.2 (3)
O1—Ca2—Ca1	42.59 (11)	C17A—C16—Ca2	171.1 (5)
O5—Ca2—Ca1	35.85 (11)	C16—C17A—N4	109.8 (6)
O8—Ca2—Ca1	122.59 (13)	C16—C17A—C18A	109.4 (7)
O7—Ca2—Ca1	93.67 (11)	N4—C17A—C18A	103.0 (6)
O6—Ca2—Ca1	84.68 (10)	C16—C17A—H17A	111.4
C16—Ca2—Ca1	108.72 (14)	N4—C17A—H17A	111.4
C11—Ca2—Ca1	60.42 (13)	C18A—C17A—H17A	111.4
O9—Ca2—Ca1^ii^	52.50 (12)	C19A—C18A—C17A	103.0 (8)
O4—Ca2—Ca1^ii^	129.19 (14)	C19A—C18A—H18A	111.2
O11—Ca2—Ca1^ii^	109.46 (14)	C17A—C18A—H18A	111.2
O1—Ca2—Ca1^ii^	148.63 (13)	C19A—C18A—H18B	111.2
O5—Ca2—Ca1^ii^	84.99 (11)	C17A—C18A—H18B	111.2
O8—Ca2—Ca1^ii^	36.66 (11)	H18A—C18A—H18B	109.1
O7—Ca2—Ca1^ii^	87.60 (11)	C18A—C19A—C20A	99.3 (8)
O6—Ca2—Ca1^ii^	37.70 (10)	C18A—C19A—H19A	111.9
C16—Ca2—Ca1^ii^	62.21 (14)	C20A—C19A—H19A	111.9
C11—Ca2—Ca1^ii^	61.01 (13)	C18A—C19A—H19B	111.9
Ca1—Ca2—Ca1^ii^	120.49 (4)	C20A—C19A—H19B	111.9
C1—O1—Ca2	171.9 (4)	H19A—C19A—H19B	109.6
C1—O1—Ca1	88.5 (4)	N4—C20A—C19A	104.6 (6)
Ca2—O1—Ca1	99.45 (17)	N4—C20A—H20A	110.8
C1—O2—Ca1	95.2 (4)	C19A—C20A—H20A	110.8
C6—O3—Ca1	138.5 (5)	N4—C20A—H20B	110.8
C6—O4—Ca2	129.8 (4)	C19A—C20A—H20B	110.8
C11—O5—Ca1	151.4 (4)	H20A—C20A—H20B	108.9
C11—O5—Ca2	102.0 (4)	C19B—C18B—H18C	111.1
Ca1—O5—Ca2	106.21 (18)	C19B—C18B—H18D	111.1
C11—O6—Ca1^ii^	160.2 (5)	H18C—C18B—H18D	109.1
C11—O6—Ca2	85.0 (4)	C18B—C19B—H19C	112.0
Ca1^ii^—O6—Ca2	95.17 (16)	C18B—C19B—H19D	112.0
C16—O7—Ca2	91.1 (4)	H19C—C19B—H19D	109.6
C16—O8—Ca1^ii^	155.6 (4)	O10—C21—O9	124.6 (6)
C16—O8—Ca2	93.5 (4)	O10—C21—C22	118.0 (6)
Ca1^ii^—O8—Ca2	104.04 (17)	O9—C21—C22	117.3 (6)
C21—O9—Ca2	153.0 (4)	O10—C21—Ca1^ii^	47.5 (3)
C21—O9—Ca1^ii^	78.5 (4)	O9—C21—Ca1^ii^	77.6 (4)
Ca2—O9—Ca1^ii^	89.92 (15)	C22—C21—Ca1^ii^	164.5 (5)
C21—O10—Ca1^ii^	110.1 (4)	N5—C22—C21	111.1 (5)
Ca2—O11—H11B	117 (5)	N5—C22—C23	102.9 (6)
Ca2—O11—H11A	115 (6)	C21—C22—C23	117.9 (6)
H11B—O11—H11A	111 (8)	N5—C22—H22	108.2
H12A—O12—H12B	92 (3)	C21—C22—H22	108.2
H13B—O13—H13A	91 (4)	C23—C22—H22	108.2
C5A—N1—C2	112.5 (9)	C22—C23—C24	105.0 (7)
C2—N1—C5B	103.4 (8)	C22—C23—H23A	110.7
C5A—N1—H1A	109.1	C24—C23—H23A	110.7
C2—N1—H1A	109.1	C22—C23—H23B	110.7
C5A—N1—H1B	109.1	C24—C23—H23B	110.7
C2—N1—H1B	109.1	H23A—C23—H23B	108.8
H1A—N1—H1B	107.8	C25—C24—C23	107.4 (6)
C7—N2—C10	108.6 (6)	C25—C24—H24A	110.2
C7—N2—H2A	110.0	C23—C24—H24A	110.2
C10—N2—H2A	110.0	C25—C24—H24B	110.2
C7—N2—H2B	110.0	C23—C24—H24B	110.2
C10—N2—H2B	110.0	H24A—C24—H24B	108.5
H2A—N2—H2B	108.3	N5—C25—C24	103.8 (7)
C12—N3—C15A	109.0 (5)	N5—C25—H25A	111.0
C12—N3—H3A	109.9	C24—C25—H25A	111.0
C15A—N3—H3A	109.9	N5—C25—H25B	111.0
C12—N3—H3B	109.9	C24—C25—H25B	111.0
C15A—N3—H3B	109.9	H25A—C25—H25B	109.0
H3A—N3—H3B	108.3		

Symmetry codes: (i) −*x*+1, *y*−1/2, −*z*+1/2; (ii) −*x*+1, *y*+1/2, −*z*+1/2.

### (1) catena-poly[[aqua-µ3-L-proline-tetra-µ2-L-proline-dicalcium] tetraiodide 1.7-hydrate] Hydrogen-bond geometry (Å, º)

**Table:**

*D*—H···*A*	*D*—H	H···*A*	*D*···*A*	*D*—H···*A*
N1—H1*A*···I3^iii^	0.99	2.60	3.526 (7)	155
N1—H1*B*···O11	0.99	2.10	2.996 (9)	150
N2—H2*A*···I1	0.99	2.75	3.603 (5)	145
N2—H2*B*···I4*A*	0.99	2.53	3.400 (6)	146
N3—H3*A*···O2^ii^	0.99	1.94	2.829 (7)	147
N3—H3*B*···I2	0.99	2.61	3.447 (5)	143
N4—H4*A*···O12	0.99	1.80	2.750 (8)	159
N4—H4*B*···I3^iii^	0.99	2.92	3.674 (6)	133
N5—H5*A*···I1	0.99	2.74	3.627 (6)	149
N5—H5*B*···I3	0.99	2.62	3.478 (6)	146
O11—H11*A*···I1	0.84 (6)	2.56 (6)	3.389 (5)	171 (7)
O11—H11*B*···I2^ii^	0.83 (7)	2.71 (7)	3.524 (6)	168 (5)
O12—H12*A*···O10^iii^	0.83 (4)	2.06 (5)	2.732 (7)	137 (4)
O12—H12*B*···O2^iv^	0.83 (3)	2.54 (4)	3.339 (8)	162 (7)
O13—H13*A*···I4*A*^ii^	0.85 (11)	2.90 (11)	3.703 (11)	159 (10)
O13—H13*B*···I4*A*^v^	0.85 (8)	2.76 (8)	3.598 (11)	172 (10)

Symmetry codes: (ii) −*x*+1, *y*+1/2, −*z*+1/2; (iii) *x*+1, *y*, *z*; (iv) −*x*+2, *y*+1/2, −*z*+1/2; (v) −*x*+3/2, −*y*+1, *z*−1/2.

### (2) catena-Poly[[diaquadi-m2-DL-proline-calcium] diiodide] Crystal data

**Table:**

[Ca(C_5_H_9_NO_2_)_2_(H_2_O)_2_]I_2_	*Z* = 2
*M**_r_* = 560.17	*F*(000) = 540
Triclinic, *P*1	*D*_x_ = 2.084 Mg m^−^^3^
*a* = 7.958 (7) Å	Mo *K*α radiation, λ = 0.71073 Å
*b* = 9.080 (8) Å	Cell parameters from 1555 reflections
*c* = 13.591 (11) Å	θ = 2.4–23.8°
α = 105.757 (10)°	µ = 3.84 mm^−^^1^
β = 104.501 (11)°	*T* = 100 K
γ = 97.911 (12)°	Plate, yellow
*V* = 892.5 (13) Å^3^	0.22 × 0.13 × 0.05 mm

### (2) catena-Poly[[diaquadi-m2-DL-proline-calcium] diiodide] Data collection

**Table:**

Bruker D8 with APEX CCD area detector and Incoatec microsource diffractometer	2603 reflections with *I* > 2σ(*I*)
ω scans	*R*_int_ = 0.079
Absorption correction: multi-scan (*SADABS*; Bruker, 2008)	θ_max_ = 26.1°, θ_min_ = 2.4°
*T*_min_ = 0.447, *T*_max_ = 0.745	*h* = −9→9
8805 measured reflections	*k* = −11→11
3533 independent reflections	*l* = −16→16

### (2) catena-Poly[[diaquadi-m2-DL-proline-calcium] diiodide] Refinement

**Table:**

Refinement on *F*^2^	29 restraints
Least-squares matrix: full	Hydrogen site location: mixed
*R*[*F*^2^ > 2σ(*F*^2^)] = 0.047	H atoms treated by a mixture of independent and constrained refinement
*wR*(*F*^2^) = 0.116	*w* = 1/[σ^2^(*F*_o_^2^) + (0.020*P*)^2^] where *P* = (*F*_o_^2^ + 2*F*_c_^2^)/3
*S* = 1.02	(Δ/σ)_max_ = 0.001
3533 reflections	Δρ_max_ = 1.05 e Å^−^^3^
214 parameters	Δρ_min_ = −2.20 e Å^−^^3^

### (2) catena-Poly[[diaquadi-m2-DL-proline-calcium] diiodide] Special details

**Table:**

Geometry. All e.s.d.'s (except the e.s.d. in the dihedral angle between two l.s. planes) are estimated using the full covariance matrix. The cell e.s.d.'s are taken into account individually in the estimation of e.s.d.'s in distances, angles and torsion angles; correlations between e.s.d.'s in cell parameters are only used when they are defined by crystal symmetry. An approximate (isotropic) treatment of cell e.s.d.'s is used for estimating e.s.d.'s involving l.s. planes.

### (2) catena-Poly[[diaquadi-m2-DL-proline-calcium] diiodide] Fractional atomic coordinates and isotropic or equivalent isotropic displacement parameters (Å^2^)

**Table:**

	*x*	*y*	*z*	*U*_iso_*/*U*_eq_	
Ca1	0.7566 (2)	0.11114 (16)	0.03989 (10)	0.0214 (3)	
O1	0.4901 (7)	0.0237 (6)	0.1072 (4)	0.0294 (12)	
O2	0.7073 (7)	0.2219 (6)	0.2165 (4)	0.0302 (12)	
O5	0.6524 (7)	0.3423 (6)	0.0331 (4)	0.0248 (12)	
H1W	0.576 (8)	0.367 (9)	0.063 (6)	0.030*	
H2W	0.625 (10)	0.331 (9)	−0.032 (2)	0.030*	
O6	1.0260 (8)	0.2610 (7)	0.1713 (4)	0.0360 (14)	
H3W	1.125 (6)	0.313 (7)	0.169 (6)	0.043*	
H4W	1.028 (12)	0.256 (10)	0.232 (3)	0.043*	
O3	0.8632 (7)	−0.1067 (6)	0.0374 (4)	0.0303 (13)	
O4	1.0911 (7)	−0.2226 (6)	0.0632 (4)	0.0305 (13)	
N2	0.9542 (9)	−0.4002 (8)	0.1626 (5)	0.0257 (15)	
H2A	1.053 (7)	−0.415 (9)	0.153 (6)	0.031*	
H2B	0.896 (10)	−0.491 (6)	0.120 (5)	0.031*	
N1	0.5637 (9)	0.2912 (7)	0.3744 (5)	0.0250 (14)	
H1A	0.480 (8)	0.329 (9)	0.393 (6)	0.030*	
H1B	0.625 (9)	0.362 (7)	0.358 (6)	0.030*	
C10	0.8593 (11)	−0.1694 (9)	0.2437 (6)	0.0308 (18)	
H8	0.8894	−0.0586	0.2463	0.037*	
H9	0.7423	−0.1900	0.2563	0.037*	
C9	1.0000 (12)	−0.2034 (10)	0.3254 (6)	0.038 (2)	
H10	0.9776	−0.1760	0.3959	0.045*	
H11	1.1193	−0.1437	0.3336	0.045*	
C8	0.9858 (11)	−0.3790 (9)	0.2807 (5)	0.0312 (19)	
H12	1.0970	−0.4091	0.3119	0.037*	
H13	0.8848	−0.4402	0.2933	0.037*	
C7	0.8560 (10)	−0.2808 (8)	0.1353 (5)	0.0246 (16)	
H14	0.7302	−0.3332	0.0914	0.030*	
C6	0.9462 (10)	−0.1969 (8)	0.0726 (5)	0.0240 (16)	
C5	0.4773 (11)	0.0135 (9)	0.3293 (6)	0.0292 (18)	
H1	0.3804	−0.0009	0.3617	0.035*	
H2	0.4713	−0.0866	0.2749	0.035*	
C4	0.6589 (12)	0.0713 (9)	0.4148 (6)	0.034 (2)	
H3	0.6670	0.0152	0.4683	0.041*	
H4	0.7560	0.0567	0.3821	0.041*	
C3	0.6678 (11)	0.2433 (9)	0.4655 (5)	0.0287 (18)	
H5	0.7926	0.3041	0.4943	0.034*	
H6	0.6129	0.2594	0.5243	0.034*	
C2	0.4668 (11)	0.1463 (8)	0.2801 (5)	0.0251 (15)	
H7	0.3402	0.1509	0.2497	0.030*	
C1	0.5620 (10)	0.1303 (8)	0.1964 (5)	0.0211 (12)	
I1	0.40641 (6)	0.52553 (5)	0.18903 (3)	0.02488 (16)	
I2	0.18089 (7)	0.31320 (6)	0.44926 (3)	0.02940 (17)	

### (2) catena-Poly[[diaquadi-m2-DL-proline-calcium] diiodide] Atomic displacement parameters (Å^2^)

**Table:**

	*U*^11^	*U*^22^	*U*^33^	*U*^12^	*U*^13^	*U*^23^
Ca1	0.0248 (8)	0.0207 (7)	0.0185 (6)	0.0044 (6)	0.0025 (6)	0.0100 (5)
O1	0.034 (3)	0.032 (2)	0.0183 (17)	0.007 (2)	0.0011 (17)	0.0083 (16)
O2	0.029 (2)	0.034 (3)	0.025 (2)	0.0015 (19)	0.0045 (18)	0.0125 (19)
O5	0.033 (3)	0.025 (3)	0.019 (2)	0.010 (2)	0.008 (2)	0.011 (2)
O6	0.034 (4)	0.046 (4)	0.023 (2)	−0.003 (3)	0.006 (2)	0.011 (2)
O3	0.037 (3)	0.026 (3)	0.034 (3)	0.014 (3)	0.009 (2)	0.018 (2)
O4	0.032 (3)	0.040 (3)	0.032 (3)	0.013 (3)	0.013 (2)	0.023 (2)
N2	0.032 (4)	0.026 (3)	0.020 (3)	0.012 (3)	0.005 (3)	0.009 (2)
N1	0.032 (4)	0.022 (3)	0.023 (3)	0.007 (3)	0.008 (3)	0.010 (2)
C10	0.041 (5)	0.026 (4)	0.032 (4)	0.010 (4)	0.017 (4)	0.012 (3)
C9	0.039 (5)	0.048 (5)	0.018 (3)	0.001 (4)	0.002 (3)	0.010 (3)
C8	0.038 (5)	0.034 (4)	0.025 (3)	0.011 (4)	0.008 (3)	0.016 (3)
C7	0.027 (4)	0.024 (4)	0.026 (3)	0.007 (3)	0.006 (3)	0.014 (3)
C6	0.026 (4)	0.020 (4)	0.021 (3)	0.004 (3)	0.005 (3)	0.002 (3)
C5	0.031 (5)	0.027 (4)	0.028 (4)	−0.002 (4)	0.010 (3)	0.009 (3)
C4	0.042 (5)	0.035 (5)	0.028 (4)	0.009 (4)	0.006 (3)	0.017 (3)
C3	0.030 (5)	0.038 (5)	0.019 (3)	0.012 (4)	0.005 (3)	0.011 (3)
C2	0.033 (4)	0.018 (4)	0.024 (2)	0.002 (3)	0.008 (3)	0.008 (2)
C1	0.025 (2)	0.023 (3)	0.0176 (18)	0.0091 (18)	0.0015 (16)	0.0138 (16)
I1	0.0270 (3)	0.0256 (3)	0.0244 (2)	0.0070 (2)	0.00549 (19)	0.01326 (19)
I2	0.0328 (3)	0.0318 (3)	0.0246 (2)	0.0078 (2)	0.0083 (2)	0.0107 (2)

### (2) catena-Poly[[diaquadi-m2-DL-proline-calcium] diiodide] Geometric parameters (Å, º)

**Table:**

Ca1—O1	2.621 (6)	N1—C3	1.512 (8)
Ca1—O2	2.489 (6)	N1—H1A	0.86 (4)
Ca1—O4^i^	2.396 (5)	N1—H1B	0.86 (4)
Ca1—O5	2.376 (5)	C10—C9	1.496 (10)
Ca1—O6	2.365 (6)	C10—C7	1.536 (10)
Ca1—O1^ii^	2.323 (5)	C10—H8	0.9900
Ca1—O3	2.252 (5)	C10—H9	0.9900
Ca1—Ca1^i^	4.829 (4)	C9—C8	1.521 (11)
Ca1—Ca1^ii^	4.032 (4)	C9—H10	0.9900
Ca1—C1	2.911 (8)	C9—H11	0.9900
Ca1—C6^i^	3.238 (8)	C8—H12	0.9900
O1—C1	1.263 (8)	C8—H13	0.9900
O1—Ca1^ii^	2.323 (5)	C7—C6	1.527 (10)
O2—C1	1.248 (9)	C7—H14	1.0000
O5—H1W	0.83 (2)	C6—Ca1^i^	3.238 (8)
O5—H2W	0.83 (2)	C5—C4	1.527 (10)
O6—H3W	0.876 (17)	C5—C2	1.531 (10)
O6—H4W	0.84 (2)	C5—H1	0.9900
O3—C6	1.241 (8)	C5—H2	0.9900
O4—C6	1.237 (9)	C4—C3	1.510 (11)
O4—Ca1^i^	2.396 (5)	C4—H3	0.9900
N2—C7	1.493 (9)	C4—H4	0.9900
N2—C8	1.515 (9)	C3—H5	0.9900
N2—H2A	0.85 (4)	C3—H6	0.9900
N2—H2B	0.85 (4)	C2—C1	1.505 (10)
N1—C2	1.509 (9)	C2—H7	1.0000
			
O3—Ca1—O1^ii^	92.68 (19)	H3W—O6—H4W	114 (8)
O3—Ca1—O6	88.9 (2)	C6—O3—Ca1	158.2 (5)
O1^ii^—Ca1—O6	171.4 (2)	C6—O4—Ca1^i^	122.8 (4)
O3—Ca1—O5	176.2 (2)	C7—N2—C8	108.4 (6)
O1^ii^—Ca1—O5	86.69 (18)	C7—N2—H2A	127 (6)
O6—Ca1—O5	91.2 (2)	C8—N2—H2A	103 (5)
O3—Ca1—O4^i^	102.3 (2)	C7—N2—H2B	109 (5)
O1^ii^—Ca1—O4^i^	93.50 (19)	C8—N2—H2B	115 (5)
O6—Ca1—O4^i^	77.94 (19)	H2A—N2—H2B	94 (7)
O5—Ca1—O4^i^	73.95 (19)	C2—N1—C3	109.3 (5)
O3—Ca1—O2	109.18 (18)	C2—N1—H1A	104 (5)
O1^ii^—Ca1—O2	118.14 (19)	C3—N1—H1A	108 (5)
O6—Ca1—O2	69.1 (2)	C2—N1—H1B	114 (5)
O5—Ca1—O2	74.40 (17)	C3—N1—H1B	114 (5)
O4^i^—Ca1—O2	133.12 (19)	H1A—N1—H1B	107 (7)
O3—Ca1—O1	94.58 (19)	C9—C10—C7	105.1 (6)
O1^ii^—Ca1—O1	70.85 (19)	C9—C10—H8	110.7
O6—Ca1—O1	117.43 (18)	C7—C10—H8	110.7
O5—Ca1—O1	88.80 (18)	C9—C10—H9	110.7
O4^i^—Ca1—O1	157.57 (16)	C7—C10—H9	110.7
O2—Ca1—O1	50.92 (16)	H8—C10—H9	108.8
O3—Ca1—C1	103.14 (19)	C10—C9—C8	104.3 (6)
O1^ii^—Ca1—C1	94.8 (2)	C10—C9—H10	110.9
O6—Ca1—C1	93.0 (2)	C8—C9—H10	110.9
O5—Ca1—C1	80.70 (18)	C10—C9—H11	110.9
O4^i^—Ca1—C1	152.77 (19)	C8—C9—H11	110.9
O2—Ca1—C1	25.20 (17)	H10—C9—H11	108.9
O1—Ca1—C1	25.71 (17)	N2—C8—C9	101.0 (6)
O3—Ca1—C6^i^	85.3 (2)	N2—C8—H12	111.6
O1^ii^—Ca1—C6^i^	102.18 (19)	C9—C8—H12	111.6
O6—Ca1—C6^i^	69.55 (19)	N2—C8—H13	111.6
O5—Ca1—C6^i^	91.14 (19)	C9—C8—H13	111.6
O4^i^—Ca1—C6^i^	18.73 (16)	H12—C8—H13	109.4
O2—Ca1—C6^i^	135.61 (18)	N2—C7—C6	110.1 (6)
O1—Ca1—C6^i^	173.02 (16)	N2—C7—C10	105.0 (5)
C1—Ca1—C6^i^	160.72 (19)	C6—C7—C10	112.6 (6)
O3—Ca1—Ca1^ii^	94.52 (16)	N2—C7—H14	109.7
O1^ii^—Ca1—Ca1^ii^	37.88 (14)	C6—C7—H14	109.7
O6—Ca1—Ca1^ii^	150.35 (15)	C10—C7—H14	109.7
O5—Ca1—Ca1^ii^	87.31 (15)	O4—C6—O3	127.2 (7)
O4^i^—Ca1—Ca1^ii^	129.49 (14)	O4—C6—C7	118.5 (6)
O2—Ca1—Ca1^ii^	82.06 (14)	O3—C6—C7	114.2 (7)
O1—Ca1—Ca1^ii^	32.97 (10)	O4—C6—Ca1^i^	38.4 (3)
C1—Ca1—Ca1^ii^	57.49 (15)	O3—C6—Ca1^i^	88.8 (5)
C6^i^—Ca1—Ca1^ii^	140.06 (13)	C7—C6—Ca1^i^	156.9 (5)
O3—Ca1—H2W	159.2 (9)	C4—C5—C2	102.8 (6)
O1^ii^—Ca1—H2W	74.4 (14)	C4—C5—H1	111.2
O6—Ca1—H2W	101.6 (15)	C2—C5—H1	111.2
O5—Ca1—H2W	17.6 (9)	C4—C5—H2	111.2
O4^i^—Ca1—H2W	63.2 (15)	C2—C5—H2	111.2
O2—Ca1—H2W	91.5 (10)	H1—C5—H2	109.1
O1—Ca1—H2W	96.4 (16)	C3—C4—C5	103.7 (6)
C1—Ca1—H2W	94.3 (13)	C3—C4—H3	111.0
C6^i^—Ca1—H2W	81.7 (15)	C5—C4—H3	111.0
Ca1^ii^—Ca1—H2W	85.2 (17)	C3—C4—H4	111.0
O3—Ca1—H4W	85.8 (18)	C5—C4—H4	111.0
O1^ii^—Ca1—H4W	172.4 (12)	H3—C4—H4	109.0
O6—Ca1—H4W	16.2 (12)	C4—C3—N1	103.9 (5)
O5—Ca1—H4W	95.3 (18)	C4—C3—H5	111.0
O4^i^—Ca1—H4W	94.1 (12)	N1—C3—H5	111.0
O2—Ca1—H4W	55.7 (15)	C4—C3—H6	111.0
O1—Ca1—H4W	101.8 (12)	N1—C3—H6	111.0
C1—Ca1—H4W	78.3 (13)	H5—C3—H6	109.0
C6^i^—Ca1—H4W	85.2 (12)	C1—C2—N1	109.3 (6)
Ca1^ii^—Ca1—H4W	134.7 (12)	C1—C2—C5	111.5 (6)
H2W—Ca1—H4W	109 (2)	N1—C2—C5	103.2 (5)
C1—O1—Ca1^ii^	150.9 (5)	C1—C2—H7	110.9
C1—O1—Ca1	90.1 (5)	N1—C2—H7	110.9
Ca1^ii^—O1—Ca1	109.15 (19)	C5—C2—H7	110.9
C1—O2—Ca1	96.7 (4)	O2—C1—O1	122.3 (7)
Ca1—O5—H1W	120 (6)	O2—C1—C2	119.6 (6)
Ca1—O5—H2W	102 (5)	O1—C1—C2	118.1 (7)
H1W—O5—H2W	115 (8)	O2—C1—Ca1	58.1 (4)
Ca1—O6—H3W	134 (5)	O1—C1—Ca1	64.2 (4)
Ca1—O6—H4W	112 (6)	C2—C1—Ca1	177.7 (5)

Symmetry codes: (i) −*x*+2, −*y*, −*z*; (ii) −*x*+1, −*y*, −*z*.

### (2) catena-Poly[[diaquadi-m2-DL-proline-calcium] diiodide] Hydrogen-bond geometry (Å, º)

**Table:**

*D*—H···*A*	*D*—H	H···*A*	*D*···*A*	*D*—H···*A*
N1—H1*A*···I2	0.85 (7)	2.67 (7)	3.459 (8)	154 (7)
N1—H1*B*···I1	0.86 (7)	3.33 (7)	3.809 (7)	118 (6)
N1—H1*B*···I2^iii^	0.86 (7)	3.24 (7)	3.695 (7)	116 (5)
N2—H2*A*···I1^iv^	0.85 (6)	2.88 (6)	3.700 (8)	161 (7)
N2—H2*B*)···O5^v^	0.86 (6)	2.13 (7)	2.904 (9)	151 (7)
O5—H1*W*···I1	0.84 (7)	2.68 (7)	3.486 (6)	163 (6)
O5—H2*W*···I1^vi^	0.83 (3)	2.77 (6)	3.491 (6)	147 (7)
O6—H3*W*···I1^vii^	0.87 (6)	2.65 (6)	3.509 (7)	167 (5)
O6—H4*W*···I2^vii^	0.84 (5)	2.77 (4)	3.543 (6)	155 (7)

Symmetry codes: (iii) −*x*+1, −*y*+1, −*z*+1; (iv) *x*+1, *y*−1, *z*; (v) *x*, *y*−1, *z*; (vi) −*x*+1, −*y*+1, −*z*; (vii) *x*+1, *y*, *z*.

## References

[bb1] Batten, S. R., Neville, S. M. & Turner, D. R. (2008). *Coordination Polymers: Design, Analysis and Application*, pp. 172–178, 202–212. London: Royal Society of Chemistry.

[bb2] Bruker (2008). *SMART*, *SAINT-Plus* and *SADABS*. Bruker AXS Inc., Madison, Wisconsin, USA.

[bb3] Fleck, M. & Petrosyan, A. M. (2014). *Salts of amino acids: Crystallization, Structure and Properties*. Switzerland: Springer International Publishing.

[bb4] Groom, C. R. & Allen, F. H. (2014). *Angew. Chem. Int. Ed.* **53**, 662–671. 10.1002/anie.201306438 24382699

[bb5] Ito, T., Marumo, F. & Saito, Y. (1971). *Acta Cryst.* B**27**, 1062–1066.

[bb6] Kato, M., Hayashi, M., Fujihara, T. & Nagasawa, A. (2008). *Acta Cryst.* E**64**, m684. 10.1107/S1600536808010246 PMC2961198 21202221

[bb7] Kim, E. E., Sicignano, A. & Eriks, K. (1985). *J. Am. Chem. Soc.* **107**, 6042–6046.

[bb8] Lamberts, K., Möller, A. & Englert, U. (2014*a*). *Acta Cryst.* B**70**, 989–998. 10.1107/S2052520614021398 25449622

[bb9] Lamberts, K., Porsche, S., Hentschel, B., Kuhlen, T. & Englert, U. (2014*b*). *CrystEngComm*, **16**, 3305–3311.

[bb10] Lamberts, K., Şerb, M.-D. & Englert, U. (2015). *Acta Cryst.* C**71**, 311–317. 10.1107/S2053229615005008 25836292

[bb11] Macrae, C. F., Bruno, I. J., Chisholm, J. A., Edgington, P. R., McCabe, P., Pidcock, E., Rodriguez-Monge, L., Taylor, R., van de Streek, J. & Wood, P. A. (2008). *J. Appl. Cryst.* **41**, 466–470.

[bb12] Magill, C. P., Floriani, C., Chiesi-Villa, A. & Rizzoli, C. (1993). *Inorg. Chem.* **32**, 2729–2735.

[bb13] Marandi, F. & Shahbakhsh, N. (2007). *J. Coord. Chem.* **60**, 2589–2595.

[bb14] Mathieson, A. & Welsh, H. K. (1952). *Acta Cryst.* **5**, 599–604.

[bb15] Mikhalyova, E. A., Kolotilov, S. V., Cador, O., Pointillart, F., Golhen, S., Ouahab, L. & Pavlishchuk, V. V. (2010). *Inorg. Chim. Acta*, **363**, 3453–3460.

[bb16] Oki, H. & Yoneda, H. (1981). *Inorg. Chem.* **20**, 3875–3879.

[bb17] Parsons, S. & Flack, H. (2004). *Acta Cryst.* A**60**, s61.

[bb18] Schmidbaur, H., Kiprof, P., Kumberger, O. & Riede, J. (1991). *Chem. Ber.* **124**, 1083–1087.

[bb19] Sheldrick, G. M. (2008). *Acta Cryst.* A**64**, 112–122. 10.1107/S0108767307043930 18156677

[bb20] Sheldrick, G. M. (2015). *Acta Cryst.* C**71**, 3–8.

